# Muslim parents’ beliefs and factors influencing complete immunization of children aged 0–5 years in a Thai rural community: a qualitative study

**DOI:** 10.1186/s12889-023-15273-y

**Published:** 2023-07-13

**Authors:** Taqwa Jinarong, Rattanaporn Chootong, Polathep Vichitkunakorn, Praneed Songwathana

**Affiliations:** 1 Department of Family and Preventive Medicine, Faculty of Medicine, Prince of Songkla University, 15 Karnjanavanich Road, Hat Yai, 90110 Songkhla, Thailand; 2 Faculty of Nursing, Prince of Songkla University, 15 Karnjanavanich Road, Hat Yai, 90110 Songkhla, Thailand

**Keywords:** Belief, Childhood vaccination, Negative factors, Positive factors, Parents

## Abstract

**Purpose:**

Vaccine-preventable diseases have decreased globally. However, measles and diphtheria outbreaks still occur in Southern Thailand, where Muslims are predominant with a documented low vaccine coverage. The purpose of this study was to investigate Muslim parents’ beliefs and factors influencing them to complete immunization of children aged 0–5 years in Y.L. province, Thailand.

**Method:**

A descriptive qualitative study was conducted, using focus group discussion with 26 participants. They are parents whose children had complete or incomplete vaccination and community/religious leaders. Data were analyzed using content-analysis and triangulation method was used to ensure trustworthiness.

**Results:**

Four major themes emerged from the analysis: (1) positive vaccine beliefs, which included knowledge and awareness of vaccination, trust in vaccine efficacy, and religious beliefs; (2) positive factors influencing positive beliefs and vaccine acceptance, which were accessibility of reliable sources, and imitation of leaders and health-community-network; (3) negative vaccine beliefs, including bias in vaccine efficacy and safety, personal beliefs about sources of vaccines, and religious misconceptions regarding the value of vaccines and Halal concerns; and (4) negative factors influencing negative beliefs and refusal of vaccination, which were perception of disadvantages of vaccines spread by word-of-mouth, trust in person over empirical evidence, religious views based on self-interpretation, and lack of public information on Halal vaccines.

**Conclusion:**

Both positive and negative factors influencing complete immunization were found in this study. To enhance vaccine acceptance, health care providers should understand Muslim cultural beliefs by offering parents a chance to express their attitudes and encourage vaccination via religious leaders and community role models.

## Introduction

The most effective way of disease prevention in children is to immunize them through vaccinations. The World Health Organization (WHO) set up the Expanded Programme on Immunization (EPI) in 1970, to vaccinate children globally. Vaccine-preventable diseases have since been decreasing, because of the increasing number of children receiving routine, recommended vaccines worldwide [1, 2]. However, global vaccine coverage varies across different types of vaccines. In 2019, diphtheria, tetanus, and pertussis vaccines (DTP1) had a 90% coverage, whereas, measles vaccines had a lower coverage of 71% [3].

In Thailand, routine immunization program for children including BCG, HB, DPT, Hib, OPV, IPV, MMR, LAJE and Rota [2]. National survey in 2018, revealed that most vaccines met the 90% threshold [4]. Unfortunately, in Y.L. province, which is one of the far, Southern provinces of Thailand, children have been receiving overall immunization program at a substantially lower rate (approximately 64% from 2016 to 2020) [5].

Since 2000, although measles cases have seen a downtrend in Thailand, there have been measles epidemics causing disability and mortality every 3–5 years in the Southern provinces. Nationwide results have shown that there were 7,229 measles cases in 2018 (incidence rate 56.21 per 100,000 population). Y.H. district of Y.L. province had the highest number of measles cases in Thailand (incidence rate 746.40 per 100,000 population) [6, 7], with a documented low MMR vaccine coverage (approximately 70% from 2014 to 2018) [5]. Hence, Thailand failed to reach the goal of eradicating measles in 2020, regardless of the commitment to WHO [8]. Therefore, this location was chosen for this study.

Y.H. district of Y.L. province in Southern Thailand, where Muslims are predominant have one of the lowest coverages of overall childhood vaccines and the highest measles outbreak [5, 8]. There has been an issue that children had incomplete immunization because the Muslims mainstream refused vaccines [9, 10]. Vaccine acceptance and lifestyles of residents of Y.H. are heavily influenced by Islamic standards and religious leaders. Their characteristics are different from that of other communities in Thailand, in which people are mainly Buddhist perceiving susceptibility of vaccine-preventable diseases and not against vaccination [11], although it is seen among Malaysian people who have a similar Muslim culture [12]. Y.L. is one of three provinces adjacent to Malaysia, having some people migrating between both countries.

Various factors, including lack of knowledge, perception, belief on vaccines, have prevented parents from getting routine vaccinations for their children. A study by Mckee C et al. [13] highlighted that parents were worried about side effects of vaccines, and that they preferred disease preventions using natural methods. Moreover, a study by Lim WY et al. [12], found that Muslim parents in Malaysia distrusted efficacy of vaccines and suspected that vaccines might contain *haram* (impermissible) ingredients. Issues under the beliefs of Halal (meaning permissible or lawful in Islam, in reference to food, costume, and behavior that are of Islamic standards, as prescribed in the Shari’ah (Islamic Law)) and Haram (meaning impermissible or unlawful in Islam) [14] in relation to certificated vaccine ingredients were unexplored.

Additionally, socioeconomic factors suppress routine childhood vaccination rates in Thailand. Wanawanakorn et al. and Sornsrivichai et al. [15, 16] noted the barriers for childhood vaccination in the Southern provinces including religious beliefs and parents’ aversion to interact with healthcare providers. Additionally, the lack of reminders from medical staff, long waiting times, and parents’ tendency to forget about vaccination appointments compound the problem of incomplete vaccination [12].

Most recent studies on children’s vaccination were quantitative that focused on the negative factors and did not highlight people’s perceptions and beliefs regarding their children’s vaccination. Therefore, there is a need for qualitative studies to explore this sensitive issue in depth. Moreover, there was a lack of analysis on positive factors encouraging completed vaccination. This phenomenon is multi-faceted, which requires qualitative analysis of culture, society, and people’s beliefs in rural Muslim communities, whose members strictly follow religious traditions. Thus, this study focused on how Muslim parents’ level of knowledge, religious beliefs, and ways of life affected their decisions and attitudes toward vaccinating their children. The study will help the parents express their attitudes on vaccination. It could deepen the understanding of parents’ decision-making, specifically, the motivations and obstacles the have to vaccinate their children. Healthcare providers and policymakers can use this knowledge to create campaigns and strategies to enhance vaccination coverage.

## Materials and methods

### Study design

This was a descriptive qualitative study using focus group discussions to collect data from September to November 2021.

### Study setting and study population

The study was conducted in Y.H. district of Y.L. province in Southern Thailand, a rural Muslim community.

The key informants in this study were Muslim parents of completely, partially, or none vaccinated children aged 0–5 years, recruited from the Health Data Centre (HDC) of Y.L. province using purposive and snowball sampling technique. The other informants were religious/community leaders who regularly engage with the population and could clarify religious and social topics based on accurate evidence. All participants were contacted from village health volunteers via face-to-face invitation. They could communicate in Thai and provided consent to participate in this study. Parents whose children were chronically ill, with disability, or frequently hospitalized were excluded.

In this study, the 26 participants recruited from Y.H. district consisted of 12 parents whose children received full item and dose of routine immunization program (complete vaccination; CV), seven parents whose children missed at least one dose of routine immunization program (incomplete vaccination; IV), and seven community/religious leaders (L). Recruitment of participants was continued until data saturation was reached.

### Study tools

A semi-structured interview guide was used to include questions related to any preconceptions and pre-existing knowledge of diseases and vaccines, experiences with antenatal care and children’s vaccination, contextual influences, and perception of the infrastructure for immunization services, such as: “Why children have to get vaccinated completely?”, “Do you trust in vaccines and why?”, and “How do the family and community affect your decision-making about vaccination?” The interview guide was prepared by the research team and its quality was validated by three experts, who each had expertise in family and preventive medicine, epidemiology and research methodology, and qualitative study. A pilot study was conducted with similar participants to improve the questions before involving the actual participants, and group discussion techniques were practiced with experts on qualitative research.

### Data collection

Data were collected using focus group discussions in three separated groups. Each group was homogenous either complete vaccination, incomplete vaccination, or community/religious leaders. As the study was conducted during the COVID-19 pandemic, the Zoom application (Zoom Video Communications, Inc., San Jose, California) was used and conversations with participants were recorded. Two researchers collected data after establishing a rapport with participants by introducing the research team and the goals of this study. One researcher acted as a moderator, while another noted the participants’ body language and the atmosphere during the discussion. For contextual conversations, participants were willing to share their opinions without notable negative emotions. Each group discussion lasted for 60–90 min. Every conversation was transcribed verbatim afterwards daily, from which the team tried to understand it thoroughly and derived the findings. Finally, the team verified whether the study had reached the point of saturation.

### Data analysis

Data were analyzed using content-analysis method [17, 18]. The research team consisted of physicians and nurses working in local hospital with experience of community health and incomplete immunization, and social health researchers. Two researchers derived the main ideas from the raw data and decoded them using independent coding based on a pre-existing conceptual framework which was organized into themes and sub-themes. This conceptual framework was concluded from Health Belief Model (Rosenstock, 1974) [19] and Social Determinants of Health (WHO, 2010) [20] which explain health-related behavior and socioeconomic context. Reassembling codes showed the findings in the form of a chain diagram. The preliminary conclusions were outlined and data was continually collected to reach the final conclusions. Finally, the data was verified using triangulation methods by presenting a clear description of the study’s methodological processes as a review of data codes and the findings were confirmed by two researchers.

### Ethical issues

Participants’ privacy was ensured and they were asked to provide written informed consent before any focus group discussion. While discussing, any participant could turn on or off their video camera and the data collection team recorded a video clip only after receiving the participant’s consent. Only two researchers were able to access the data and video recordings using a password. The project was reviewed by the Institutional Review Board, Faculty of Medicine, Prince of Songkla University (no. 64-142-9-4).

## Results

### Participant characteristics

Data were saturated when the total number of participants reached 26. In the parents’ group, participants were all women while all leaders were men. Average age of parents was 27.7 ± 3.7 years and 34.3 ± 3.5 years for the CV and IV groups, respectively. Most parents had high school-level education, worked in the commerce industry, and had two children. Twelve parents accepted all vaccines, while seven parents hesitated for some and one parent refused all vaccines (Table 1). In the L group, two community leaders and five religious leaders had been working in each village for over 10 years.

**Table 1:** Demographics of parents

Participant code	Age	Occupation	Number of children	Vaccination categories
**Complete vaccination group (CV)**				
**CV1**	33	Farmer	2	Accepting of all vaccines
**CV2**	26	Commerce	3	Accepting of all vaccines
**CV3**	20	Employee	1	Accepting of all vaccines
**CV4**	25	Commerce	2	Accepting of all vaccines
**CV5**	29	Government officer	2	Accepting of all vaccines
**CV6**	26	Commerce	2	Accepting of all vaccines
**CV7**	29	Commerce	2	Accepting of all vaccines
**CV8**	29	Employee	2	Accepting of all vaccines
**CV9**	31	Government officer	2	Accepting of all vaccines
**CV10**	33	Housewife	2	Accepting of all vaccines
**CV11**	25	Company officer	2	Accepting of all vaccines
**CV12**	26	Housewife	2	Accepting of all vaccines
**Incomplete vaccination group (IV)**				
**IV13**	30	Farmer	2	Hesitancy of vaccines
**IV14**	31	Commerce	2	Hesitancy of vaccines
**IV15**	33	Commerce	4	Hesitancy of vaccines
**IV16**	37	Farmer	3	Hesitancy of vaccines
**IV17**	40	Farmer	2	Hesitancy of vaccines
**IV18**	36	Commerce	3	Hesitancy of vaccines
**IV19**	33	Government officer	2	Refusal of all vaccines
**Leader group (L)**				
**L20**	56	Religious leader, Commerce	-	-
**L21**	54	Religious leader, Commerce	-	-
**L22**	50	Religious leader, Farmer	-	-
**L23**	50	Religious leader, Commerce	-	-
**L24**	78	Religious leader, Farmer	-	-
**L25**	67	Mayor of subdistrict municipality	-	-
**L26**	42	Village headman	-	-

**Fig. 1:**
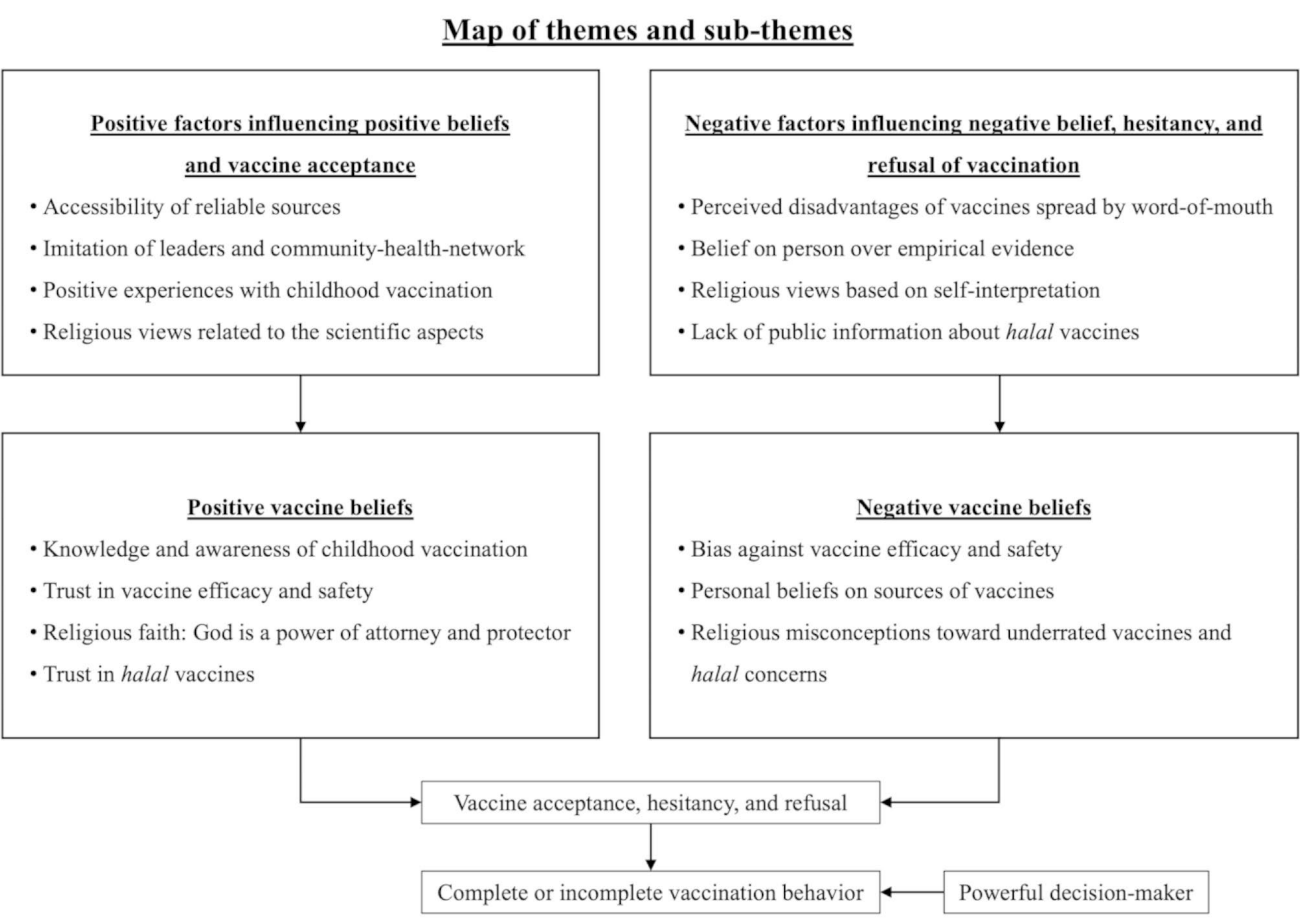
Map of themes and sub-themes of beliefs and associated factors on vaccination

From the analyses, parents’ beliefs of vaccines were found to be of multidimensional-complexity. Positive influences on positive beliefs motivated vaccine acceptance; however, negative influences on negative beliefs were barriers causing vaccine hesitancy and refusal. Major themes and sub-themes of vaccine beliefs and associated factors emerged from the analysis (Fig. 1).

### Positive beliefs of vaccines promoting vaccination behaviors

Various positive beliefs promoting parental acceptance of vaccines resulting in complete immunization were found:

#### Knowledge and awareness of childhood vaccination

Fifteen parents had knowledge of vaccine-preventable diseases and the need of immunization. They also perceived the physical and mental threats of exposing their child to illness. Participants expressed that disease prevention by vaccines was more worthy than disease treatments. They realized the vaccine schedules and were strict on vaccination appointments:

*“When my child is sick, I have to think a lot. I have to leave work and lose income. Seeking a caregiver immediately is hard, so prevention is better. Although, he was sick, but not with a serious illness…, I will not delay their vaccine schedule, because each vaccine is specific for the children’s age” (CV9)*.

#### Trust in vaccine efficacy and safety

Parents expressed that childhood vaccination campaigns were implemented with credible vaccine information published for a long time, which helped them understand that side effects were normal reactions of vaccines. Moreover, they had positive experiences such as a healthier child and no serious side effects. Twelve parents and five leaders trusted in efficacy and safety of vaccines:

*“I am 70–80% confident that vaccines provide effective immunization to prevent diseases. If my child is exposed to serious side effects such as, ‘they cannot walk’, I will accept God’s predestination, but as much as getting vaccinated previously as safe” (CV11)*.

#### Religious faith: god is a power of attorney and protector (*tawakkul*) [21]

Parents in CV group and leaders believed that illness and remedies come from God (*Allah*). Islam encourages prevention of diseases by vaccines. Hence, all Muslims are responsible for accepting the illness and finding ways of disease prevention and treatment that are within the boundaries of religious law:

*“Illness is our test destinated by Allah to be patient and remember Allah. Vaccines are Hikmah (wisdom; as the remedies in this context) from Allah for protecting us from diseases” (L21)*.

#### Trust in *halal* vaccines

Six leaders and 13 parents trusted in *halal* vaccines following a religious verdict (*fatwa*) by the chief religious leader of Thailand:

*“For halal issues, I cannot prescribe by myself, because our chief religious leader has declared the fatwa already…We are assured by our leader and accept halal vaccines.” (L23)*.

### Positive factors influencing positive beliefs and vaccine acceptance

Various factors influenced positive beliefs and vaccine acceptance, which facilitated or motivated toward complete immunization:

#### Accessibility of reliable sources of vaccination

Parents in the CV group, especially the educated, younger generation of parents, mostly sought information on vaccines from village health volunteers (VHV), after that they obtained information from social media platforms (Facebook, Line) and television. They had good judgement for information perception and believed in credible information:

*“I perceive vaccine information from VHV and healthcare providers toward a full schedule of vaccination and complete breastfeeding in 6 months.” (CV9)*.

#### Imitation of leaders and community-health-network

All leaders expressed that the leaders and community-health-networks act as role models of getting COVID-19 vaccination, it would strongly promote vaccine trust in people. Official leaders influence parents’ acceptance of vaccines resulting in complete vaccination. However, 11 parents only believed and followed the community leaders they respected:

*“Religious leaders usually announce to invite getting vaccinated. Now, most of children have complete vaccination. I trust in vaccines that are good for my child as suggestion by the chief religious’ leader” (CV11)*.

*“Religious leaders are one of the role models for getting COVID-19 vaccination in Y.H. district. They can clarify some points of Halal-Haram doubts and religious misconceptions. When leaders get vaccinated, people will get vaccinated as well. It is so different from another district that lacks leader participation.” (L25)*.

#### Positive experiences with childhood vaccination

Parents in CV group had positive experiences, such as no side effects, comfortable healthcare services, and abundant, optional vaccines at a reasonable cost. These promoted perceptions of worth in vaccines:

*“For private clinics, there are optional vaccines, but not from the government sector. I had an experience with the Rota vaccine for my first child, I noticed several issues with diarrhea, but nothing serious with the ability to take care of by myself.” (CV10)*.

#### Religious views related to the scientific aspects

Religious leaders and parents expressed that religious interpretation needed understanding of both Islamic laws and scientific knowledge. As the official committee were experts in religious and scientific knowledge, they were assured of the *halal* vaccines. Furthermore, vaccines were verified *halal* from the standard laboratory of The Halal Science Center of Chulalongkorn University, Thailand. This official statement was published on social media platforms. Therefore, people who accessed this information had more trust in *halal* vaccines:

*“The Halal Science Center of Chulalongkorn University proved and certified the vaccine ingredients, then the chief religious leader declared the fatwa as halal vaccines. We are assured of our leader and accept the official statement. If vaccines are not halal, they will certainly resist” (L23)*.

### Negative vaccine beliefs that are against vaccination

Various negative beliefs caused parents’ vaccine hesitancy/refusal to complete immunization:

#### Biases against vaccine efficacy and safety

Eight parents reported that they had always heard the news of post-vaccination side effects, such as disability, aggressive behavior, and mental retardation. The new, younger generation of parents and the elderly were concerned that serious side effects, same as the rumors, would occur. Thus, they were against overall vaccines:

*“From some news it was revealed that the vaccine has serious side effects, some children cannot walk or died after getting vaccinated…There have been vaccines for a long time, although they have been vaccinated against measles, but measles still appears in the world. People are not sure about vaccine safety and they are confused whether vaccines help in disease prevention.” (IV19)*.

#### Personal beliefs on sources of vaccines

Vaccines could be harmful for life, as reflected by a few parents. One parent believed that vaccines had been produced by some countries to destroy the global Muslim population. Another parent believed that vaccines were pathogens that made the child worse and perceived that some Muslim countries did not provide the Expanded Programme on Immunization (EPI) for children. All of the above came from misinformation from influential people.

*“I perceive the vaccines are made by foreign countries to export vaccines globally, but not to their own country…because they inject inactive pathogens into the human body causing resistance and reactions, then it can make the child worse.” (IV15)*.

#### Religious misconceptions regarding underrated vaccines and *halal* concerns

Misconceptions on vaccines were mostly reflected by the parents in the IV group. Two parents believed that every disease could happen and heal through God; truly trusting in God was a reason for vaccine refusal. Three parents and one leader doubted vaccine ingredients, because of lack of *halal* certified information:

*“I heard that vaccine is unnecessary. God give us some disease and healing also. Previous children were growth well without getting vaccinated.” (IV17)*.

*“I am not sure about halal vaccine. It may contain dangerous pathogens or impure substances” (IV15)*.

### Negative factors influencing negative belief, hesitancy/refusal of vaccination

Various negative factors influenced negative beliefs and vaccine hesitancy/refusal and were key barriers against complete immunization:

#### Perceived disadvantage of vaccines spread by word-of-mouth

Parents expressed that word-of-mouth information from the elderly and older parents have influenced them. Most of this information was unilaterally about disadvantages of vaccines, such as vaccines had serious side effects and were unnecessary for the growth of a child. Hence, parents who had higher anxiety would resist childhood vaccinations:

*“During my first pregnancy, I was worried because someone told me that a child will be sick, mentally retarded, or have aggressive behavior after being vaccinated. However, my mother-in-law was over-anxious, because she perceived that a child in a nearby community could not walk after getting vaccinated” (IV14)*.

#### Trust in people over empirical evidence

Older parents might have had no judgment for information perception, even though they were well-educated. One parent who refused vaccines trusted an influential peer over empirical evidence. They did not emphasize on credibility of information, leading to misconception and bias against vaccines:

*“Hearing from my friend as a lawyer, his child is not vaccinated, because the vaccines are made by foreign countries to export vaccines globally, but not to their own country…I don’t have enough knowledge, but my friend has more education, so I believe him. However, I don’t know which sources he gathers this information from.” (IV15)*.

#### Religious views based on self-interpretation

The *fatwa* in Islam must be based on multiple sources of knowledge from each country with world class experts being the final judges. However, one parent had unusual religious views that were based on self-interpretation, causing extreme misconceptions:

*“Religion promotes the disease treatment by good vaccines only. However, it is useless if the vaccines are not effective…vaccines are just the prevention but not the treatment, so it is not a rule in Islam” (IV19)*.

#### Lack of public information on *halal* vaccines

One leader expressed that the *halal* official report was unclear and inaccessible to all people. People who were urban, less educated people, or elderly and who did not use social media platforms, could not access this report. National religious offices should clarify the *halal* statement, so that it reaches everyone.

*“Some know halal, but some do not…I want religious leaders and halal institutes to certify halal issues. It is important to broadcast clearly that vaccines are a good thing.” (L20)*.

## Discussion

To gain a better understanding of the motivations and barriers of complete childhood immunization in Y.L. Province, Thailand, this qualitative study explored the parental vaccine beliefs and associated factors that influences their decision-making. Since majority of the population in Y.L. is comprised of Muslims with a collective social, religious, and cultural identity, their vaccine beliefs are different from those in other communities of Thailand. Most Thai people are Buddhist, who have high vaccine coverage without serious vaccine resistance [11].

The results show that different vaccine beliefs are associated with knowledge, attitude, trust, personal beliefs, and religious beliefs influencing parents’ acceptance, hesitancy, and refusal of vaccination.

### Parental vaccine acceptance with complete immunization behavior

Parental acceptance of vaccines is a key motivator to complete immunization of their children. It was found that young parents of children with completed immunization are energetic in seeking accurate vaccine information through reliable sources and consulting directly with healthcare providers. Basic simple knowledge and interesting information for open-minded parents would easily promote vaccine acceptance. However, parents who perceived a threat from vaccine-preventable diseases would accept vaccination after realizing its usefulness. Additionally, multipara parents who had positive vaccine experiences and familiarity with vaccine services would trust in vaccine efficacy and safety more. They are aware of the worthiness of prevention over acute treatment. Similar findings from previous studies showed that obtaining credible information can establish more positive attitudes toward childhood vaccinations [22–24]. However, knowledge alone may not be sufficient, as contextual influences are also important. We found that community/religious leaders who were role models, and proactive vaccination campaigns by community-health-network could stimulate and encourage parental awareness.

Islamic views are that illnesses and remedies both come from God, and all Muslims are responsible for finding ways of disease prevention and treatment. This Islamic teaching has well-enhanced the vaccine acceptance among parents who accessed credible information-sources and obtained deep understanding of Islam. Combining religious issues with scientific knowledge through sermons or community events may be effective in promoting immunization behavior.

### Parental vaccine hesitancy with incomplete immunization behavior

Parental vaccine hesitancy was influenced by various factors. Personal beliefs with biases were key barriers against complete immunization behavior. We found that parents hesitated regarding vaccination; although, they obtained massive information on this, it had mixed biased issues. They were concerned about side-effects may occur in their children, because of hearing from others with no evidence of occurring. Recent studies found the parents’ concerns regarding vaccine side-effects that it may cause mental retardation, behavioral problems, or death [13, 25]. Additionally, some parents try to justify their claims that vaccines come from other countries to destroy the Muslim population (*ummah*). A study explored the view of parents in Pakistan who rejected vaccines, because they thought vaccines were a scam made up by the United States [26]. These negative attitudes and beliefs were based on word-of-mouth spreading of vaccine disadvantages and preference to trust people over empirical evidence. The rumors have attracted the attention of the elderly (grandparents) and family heads (husbands). Some mothers who accepted vaccines but had incomplete immunization behavior, have faced resistance from those who had powerful decision-making voices on childhood vaccination in extended families. Another study similarly found that the younger generation of parents in Nepal try to communicate to make independent decisions and support for childhood vaccination within their respective families [27].

### Parental vaccine refusal with incomplete immunization behavior

Islamic teaching encourages disease prevention by immunization; it mentions that all Muslims are responsible for finding ways of disease prevention and treatment. However, some parents refuse all or some vaccines. We found that they had excuses for refusing vaccination due to religious misinterpretation. The vaccines were underrated as they just provide protection, but do not constitute a mandatory form of treatment in Islam. Moreover, there is a suspicion that the vaccines may not be permissible (*halal*) as they contain ritually impure substances (*najis*). Recent studies in Malaysia found that the reason for vaccine refusal was that the vaccines contained mixtures of pork and animal carcasses that are forbidden (*haram*) [12, 28]. However, this is not based on any religious verdict (*fatwa*) issued by the chief religious leader of Thailand or the International Islamic Fiqh Academy. Although, misinformation on *halal* issues are limited to the minority, it is important for vaccine beliefs in the Muslim community. A study in Indonesia reported a similar finding that *halal*-labeling was important to increase vaccine acceptability among Muslims [29].

For supporting positive beliefs and altering negative beliefs, offering people the chance to express their opinions regarding vaccination is essential. Using a campaign model in the context of community, such as enhancing awareness through respected role models and combining religious teaching with scientific knowledge for overcoming religious misinformation to promote completed immunization behavior, is required.

### Strengths of this study

This qualitative study focused on beliefs, barriers, and in-depth perspectives on childhood vaccination. Additionally, the research data was collected during the nationwide COVID-19 vaccination campaigns, which has been related with routine, recommended childhood vaccination. This resulted in more interesting and useful discussions and comments.

### Limitations

Online focus group discussions were conducted instead of face-to-face conversations. This may have led to unnatural communication, and hindered the observation of emotional expressions and environments. Hence, there was limited analysis from the body language of the participants. Regarding participants, all parents were mothers. Therefore, their reflection toward the role of vaccinating children may not be representative of the fathers’ or male members’ opinions who may have this responsibility. Accessibility to parents who refused all vaccines and traditional medical staff was limited; therefore, some issues of vaccination might not have been explored. Additionally, the research findings focused on local Muslims, therefore, its generalizability might be limited for the general population of Thailand and worldwide Muslims. However, these findings will be useful for improving vaccination campaign strategies in Thailand as well as in neighboring ASEAN countries.

### Suggestion

The acceptance, hesitancy, and refusal of vaccines are complex phenomena. Collecting data by focus group discussions only may not reveal all sensitive reasons. Future studies should include additional data from all refusal groups and individual in-depth interviews to focus on sensitive information that participants are unable to provide in large groups. Additionally, data should be collected from fathers, the elderly, and local and traditional medical staff to explore health values in the community.

## Conclusion

Childhood immunization in Muslim communities is strongly associated with society, religion, and culture. Studies have shown that recognizing the value of vaccines and trust in vaccine efficacy and safety are most important to parental vaccine acceptance. There are various factors that promote vaccine beliefs including access to reliable sources, imitation of good leaders, and interpretation of religion along with scientific knowledge. However, vaccine hesitancy and refusal stem from bias against vaccine efficacy and safety, and concerns about *halal* issues, which are influenced by personal credibility over empirical evidence, personal beliefs about unclear vaccine sources, and inaccessibility of public information on *halal* vaccines. Therefore, it is necessary to understand these factors in depth. Although, people should be able to express their opinion, vaccine awareness should be promoted through respected role models and provide complete vaccine information to the public.

## Data Availability

The data presented in this research are available on reasonable request from the corresponding author. The data are not publicly available due to privacy protection.

## References

[1] World Health Organization. (2022) *Immunization, Vaccines and Biologicals: National programmes and systems*. Retrieved Oct 20, 2022, from https://www.who.int/teams/immunization-vaccines-and-biologicals/essential-programme-on-immunization

[2] Chokephaibulkit K. (2019). *Book of immunization in Thailand*. Department of Disease Control. Retrieved May 15, 2022 from https://ddc.moph.go.th/uploads/publish/938420191209023015.pdf

[3] World Health Organization. Immunization dashboard: Vaccination coverage globally. Retrieved May 27., 2022 from https://immunizationdata.who.int/index.html

[4] Ministry of Public Health. (2018). Department of Disease Control. *National survey for basic and school-based immunization 2018 in Thailand*. Retrieved May 27, 2022 from https://ddc.moph.go.th/dvp/journal_detail.php?publish=10319

[5] Yala Provincial Public Health Office. (2020). *Health Data Center: HDC, immunization services, childhood vaccine coverage*. Retrieved April 10, 2022 from https://yla.hdc.moph.go.th/hdc/reports/report.php?source=epi/epi_complete.php&cat_id=4df360514655f79f13901ef1181ca1c7&id=f033ab37c30201f73f142449d037028d

[6] Ministry of Public Health. (2019). Department of Disease Control. *Thailand measles situation report 2018 to 2019*. Retrieved April 10, 2022 from https://ddc.moph.go.th/uploads/files/1112120200121090145.pdf

[7] Ministry of Public Health. (2018). Department of Disease Control. *Thailand measles database 2018*. Retrieved April 15, 2022 from https://apps-doe.moph.go.th/measles/

[8] Ministry of Public Health. (2021). Department of Disease Control. *Annual epidemiological surveillance report 2019*. Retrieved April 10, 2022 from https://ddc.moph.go.th/uploads/publish/1129820210620030431.pdf

[9] Daya S, Lillahkul N, Noin J (2018). Experience of parents of Thai Muslim Childhood aged 0–5 years in Yala Province who rejected the service of expanded program immunization with vaccine. J Department Med Serv.

[10] Puti S, Pilate A, Puti S, Talalama I, Muputi N (2018). *Health status, beliefs and health behaviors of people in the Southern border provinces* (Research Report).

[11] Yotawut M (2019). Maternal perceptions of Childhood Vaccinations: a case study of Banphaeo General Hospital Samutsakorn Province. J Nurs Health Care.

[12] Lim WY, Amar-Singh HSS, Jeganathan N, Rahmat H, Mustafa NA, Yusof M, Rahman F-S, Itam R, Chan S, N-Julia MS (2016). Exploring immunisation refusal by parents in the malaysian context. Cogent Med.

[13] McKee C, Bohannon K (2016). Exploring the reasons behind parental refusal of vaccines. J Pediatr Pharmacol Ther.

[14] Halal Monitoring Committee. (2022). *Definition of Halal*. Retrieved August 12, 2022 from https://halalhmc.org/resources/definition-of-halal/

[15] Wanawanakorn K (2016). Factors affecting coverage of measle vaccine of target group in Su-ghai Kolok District. J Prev Med Association Thail.

[16] Sornsrivichai V (2012). Parental factors affecting routine immunization in the southern provinces of Thailand.

[17] Korstjens I, Moser A (2018). Series: practical guidance to qualitative research. Part 4: trustworthiness and publishing. Eur J Gen Pract.

[18] Elo S, Kyngäs H (2008). The qualitative content analysis process. J Adv Nurs.

[19] Abraham C, Sheeran P, Ayers S, Baum A, McManus C, Newman S, Wallston K, Weinman J (2007). The health belief model. Cambridge Handbook of psychology, Health and Medicine.

[20] World Health Organization. (2010) *A conceptual framework for action on the social determinants of health social*. Retrieved Jan 13, 2023, from https://www.who.int/publications/i/item/9789241500852

[21] Noor ul Islam. (2021) *Understanding Tawakkul*. Retrieved Jan 14, 2023, from https://www.noorulislam.org.uk/understanding-tawakkul/

[22] Lim KK, Chan YY, Ani N, Rohani A, Siti Norfadhilah J, Santhi MR (2017). Complete immunization coverage and its determinants among children in Malaysia: findings from the National Health and Morbidity Survey (NHMS). Public Health.

[23] Awadh AI, Hassali MA, Al-lela OQ, Bux SH, Elkalmi RM, Hadi H (2014). Immunization knowledge and practice among malaysian parents: a questionnaire development and pilot-testing. BMC Public Health.

[24] Vannice KS, Salmon DA, Shui I, Omer SB, Kissner J, Edwards KM, Sparks R, Dekker CL, Klein NP, Gust DA (2011). Attitudes and beliefs of parents concerned about vaccines: impact of timing of immunization information. Pediatrics.

[25] Islam MS, Kamal A-HM, Kabir A, Southern DL, Khan SH, Hasan SMM, Sarkar T, Sharmin S, Das S, Roy T, Harun MDG, Chughtai AA, Homaira N, Seale H (2021). COVID-19 vaccine rumors and conspiracy theories: the need for cognitive inoculation against misinformation to improve vaccine adherence. PLoS ONE.

[26] Khan TM, Sahibzada MU (2016). Challenge to health workers and their opinions about parents refusal of oral polio vaccination in the Khyber Pakhtoon Khawa (KPK) province, Pakistan. Vaccine.

[27] Paul AM, Nepal S, Upreti K, Lohani J, Rima RN (2022). The last stretch: barriers to and facilitators of full immunization among children in Nepal’s Makwanpur District, results from a qualitative study. PLoS ONE.

[28] Ahmad NA, Jahis R, Lim KK, Rasidah R, Aris T (2017). Primary immunization among children in Malaysia: reasons for incomplete vaccination. J Vaccines Vaccination.

[29] Padmawati RS, Heywood A, Sitaresmi MN, Atthobari J, MacIntyre CR, Soenarto Y, Seale H (2019). Religious and community leaders’ acceptance of rotavirus vaccine introduction in Yogyakarta, Indonesia: a qualitative study. BMC Public Health.

